# Vascular biology 2014 in Monterey, California: celebrating 20 years of NAVBO

**DOI:** 10.1186/s13221-014-0025-4

**Published:** 2014-12-19

**Authors:** Ian W Tattersal, Henar Cuervo

**Affiliations:** Department of Obstetrics/Gynecology, Columbia University Medical Center, 1130 St. Nicholas Avenue, ICRC 916b, New York, NY 10032 USA

**Keywords:** Vascular biology, Angiogenesis, NAVBO

## Abstract

A meeting report for Vascular Biology 2014, held in Monterey, California and organized by the North American Vascular Biology Organization (NAVBO).

The North American Vascular Biology Organization celebrated its 20^th^ anniversary with the presentation of Vascular Biology 2014, which took place from October 19 to 23 at the Asilomar Conference Grounds in Pacific Grove, California. The presentations were organized into a several concurrent thematic programs, including the Developmental Vascular Biology and Genetics Workshop, organized by Brant Weinstein and Brian Black, the Vascular Inflammation Workshop, organized by Klaus Ley and Tanya Mayadas, and a number of mini-symposia sponsored by the Microcirculatory Society. In addition, there were two special sessions: Vascular Therapeutics, organized by Jan Kitajewski, and Molecular Mechanisms Underlying the Vascular Disorder Hemorrhagic Hereditary Telangiectasia (HHT), organized by Christopher W.C. Hughes and co-sponsored by Cure HHT (formerly HHT Foundation International).

With over 280 participants, 55 invited speakers, 61 selected oral presentations, and 154 posters, this meeting brought together scientists from all over the world for a week of intense and exciting interaction. It would be impossible to do justice to all of the timely and insightful presentations, but we will attempt to briefly describe some of the notable findings that were described.

The meeting began with talks from two keynote speakers. First, Gerald Crabtree discussed new methods and advances in the study of chromatin regulation. He described the role of BRG1 Associated Factors (BAFs) in the regulation of gene expression. BAFs, also called SWI/SNFs (SWItch/Sucrose NonFermentable) factors in other organisms, are nucleosome-remodeling complexes formed by several subunits. The differential assembly of these subunits is associated with different cellular fate; importantly, mutations in BAF subunits have been associated with pathological disorders. Dr. Crabtree encouraged the audience to examine this particular aspect of chromatin remodeling in the context of vascular biology.

Next, Shaun Coughlin presented an overview of the functions of Protease-Activated Receptors (PARs), and provided updates on the promising preliminary results of clinical trials of a PAR1 antagonist in the treatment of cardiovascular disease.

## Vascular and immune cell interactions

A major theme of this year’s conference was the cross-talk between the endothelium and the immune system, and the Vascular Inflammation Workshop contained many interesting presentations on this subject. Catherine C. Hedrick described the distinct behavior of a class of patrolling monocytes, which perform a unique function in the vasculature and can function to suppress tumor metastasis. Gwendolyn Randolph shared the results of a large-scale effort to genetically profile the diverse array of resident macrophages across different tissue types. As a highlight of this study, she described the functionality of one example gene, Gata6, which is uniquely expressed in peritoneal macrophages.

Luisa Iruela-Arispe spoke about the association between macrophages and the endothelium, and in particular discussed the role of this direct cell-cell interaction in mediating vascular permeability. In contrast, Jason Fish, recipient of the Springer Junior Investigator Award, demonstrated that endothelial cells and monocytes can also communicate at a distance. In his model, endothelial cells can secrete microRNA-containing microvesicles, which can be taken up by monocytes where they exert profound immunomodulatory effects.

## Developmental vascular biology and genetics

The session on vascular biology and genetics presented many new observations that have already shaped the way that we understand physiological blood vessel growth. Patric Turowski shared his discovery that permeability of the blood-brain barrier is regulated in part by polarized expression of VEGFR1 and VEGFR2 on opposite sides of endothelial cells. Shane Herbert described work in zebrafish that demonstrates the different migratory speeds of endothelial tip and stalk cells, and their progeny, within the developing intersomitic vessels. In an interesting twist, Victoria Bautch amended our understanding of the classic tip/stalk model of angiogenesis with her laboratory’s observation that multiple cells within a single sprout can stably adopt a tip cell-like morphology.

## Molecular and cellular dynamics of angiogenesis

Veronique Gebala, who actually presented in the vascular inflammation section, gave an exciting description of the ways in which cytoskeletal proteins reorganize stalk cells to induce lumen formation. Nan Qu showed us the *in vivo* effects of the novel regulator of angiogenesis, Slug (Snai2), in development and pathological angiogenesis. Using endothelial cells isolated from a breast tumor model, Andrew Dudley described an interesting autocrine bFGF/TGFβ feedback loop that controls endothelial cell fate and specification.

## Hereditary hemorrhagic telangiectasia

Sponsored by Cure HHT, this session described new advances in the search for a deeper understanding of the pathogenesis of HHT, a genetic disease that results in widespread arteriovenous malformations (AVMs). Using zebrafish, Beth Roman discussed a role for crosstalk between Notch and Alk signaling in the formation of brain AVMs. Whitney Wooderchak-Donahue discussed work employing deep sequencing to find novel rare mutations that underlie a small subset of HHT cases. Returning to the importance of immune cells in vascular biology, Paul S. Oh described a role for macrophages in the aberrant response to vascular insult in HHT that leads to AVM formation.

## Vascular therapeutics

This year’s new session on Vascular Therapeutics saw talks from academic researchers as well as pharmaceutical companies including Amgen, Eli Lily, Eisai, Genentech, and Regeneron. They discussed a number of cutting-edge therapeutic and experimental techniques, from small molecule inhibitors of angiogenic targets, to signaling pathway-activating enzymes, to high throughput methodologies for mouse gene knockouts. This session gave attendees an exciting glimpse into the ways in which basic science research is translated to the clinic, and will be reprised in next year’s meeting.

## Conclusions

Presenters at NAVBO 2014 shared an enormous volume of exciting new research, and a number of trends began to emerge as the conference went on. Although well-known for a long time now, the importance of understanding and acknowledging the existence of organ-specific cell subpopulations, and the varied and distinct mechanisms that underlie their organization and function was frequently addressed and emphasized. Though most cell types exhibit this kind of heterotypic character, it is particularly important in the endothelium of different vascular beds, and in the macrophages that contribute to context-specific angiogenesis in these tissues.

As well as exposure to a huge array of cutting-edge research, the attendees of NAVBO 2014 also had the opportunity to attend a session on publication and funding, sponsored by the Vascular Pharmacology Journal. Luisa Iruela-Arispe gave useful tips on presenting papers to journals, and Zorina Galis of the NHLBI discussed the relationship between publications, successful grant applications, and faculty appointments.

To help young investigators share their research, 26 travel awards were presented. Additionally, there were three NAVBO meritorious Awards presented: The 2014 Judah Folkman Award, presented to Tatiana V. Byzova, the 2014 Earl P. Benditt Award, presented to Jordan S. Pober, and the Springer Junior Investigator Award, presented to Jason Fish.

NAVBO’s 2014 annual conference was a resounding success, with researchers from across the globe meeting for a week of stimulating discussion, presentation, and revelation. We are already looking forward to the next meeting in October, 2015, in Hyannis, Massachusetts. The 2015 meeting will focus on vascular matrix biology, bioengineering, and the biology of signaling in the cardiovascular system. Don’t miss it!

